# Tetra­kis(4-chloro­anilinium) hexa­chlorido­stannate(IV) dichloride

**DOI:** 10.1107/S1600536812021666

**Published:** 2012-05-19

**Authors:** Benhua Zhou, Hongxia Liu

**Affiliations:** a School of Chemical and Biological Engineering, Yancheng Institute of Technology, Yancheng 224051, People’s Republic of China

## Abstract

The asymmetric unit of the title compound, (C_6_H_7_ClN)_4_[SnCl_6_]Cl_2_, comprises two 4-chloro­anilinium cations, half of an [SnCl_6_]^2−^ anion and a Cl^−^ anion. The Sn^IV^ atom, located on a special position on a twofold rotation axis, exhibits an octa­hedral environment. In the crystal, mol­ecules are linked by N—H⋯Cl hydrogen bonds between the 4-chloro­anilinium cations, [SnCl_6_]^2−^ anions and Cl^−^ anions.

## Related literature

For general background to ferroelectric metal-organic frameworks, see: Ye *et al.* (2009 ▶); Fu *et al.* (2007 ▶). For phase transitions in ferroelectric materials, see: Zhang *et al.* (2008 ▶); Zhao *et al.* (2008 ▶); Ye *et al.* (2006 ▶).
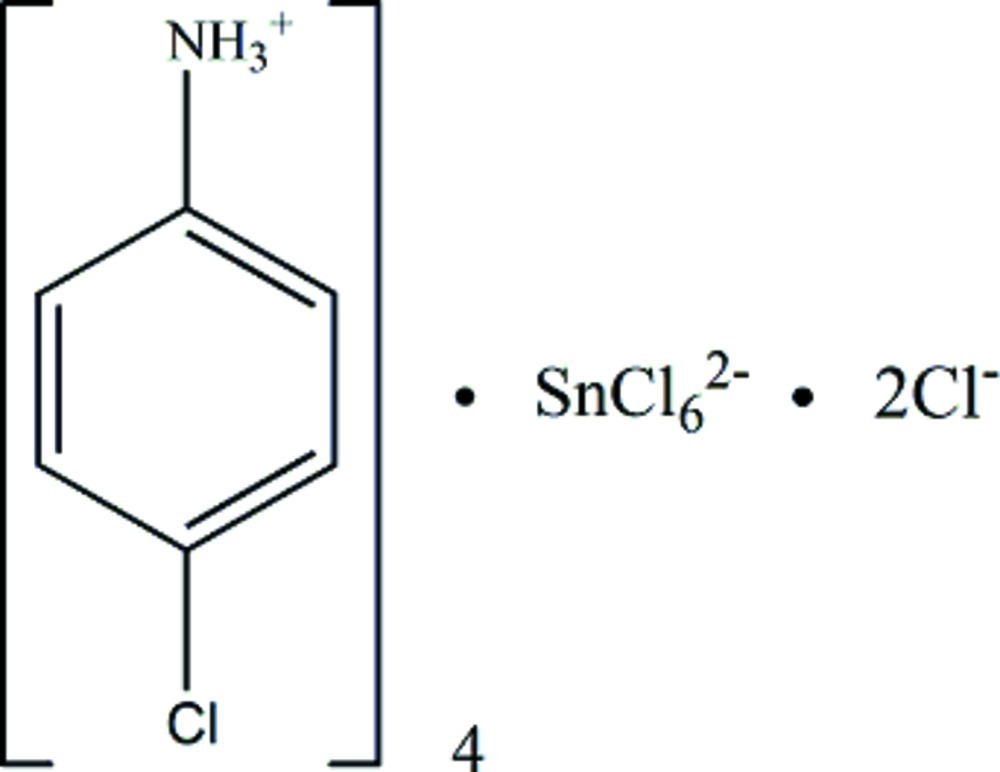


## Experimental

### 

#### Crystal data

(C_6_H_7_ClN)_4_[SnCl_6_]Cl_2_
*M* *_r_* = 916.59
Monoclinic, 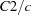

*a* = 27.855 (6) Å
*b* = 7.2061 (14) Å
*c* = 21.895 (4) Å
β = 125.03 (3)°
*V* = 3598.8 (18) Å^3^
*Z* = 4
Mo *K*α radiation
μ = 1.63 mm^−1^
*T* = 293 K
0.20 × 0.20 × 0.20 mm

#### Data collection

Rigaku SCXmini diffractometer
Absorption correction: multi-scan (*CrystalClear*; Rigaku, 2005 ▶) *T*_min_ = 0.715, *T*_max_ = 0.730
17870 measured reflections
4122 independent reflections
3581 reflections with *I* > 2σ(*I*)
*R*_int_ = 0.031

#### Refinement

*R*[*F* ^2^ > 2σ(*F* ^2^)] = 0.030
*wR*(*F* ^2^) = 0.067
*S* = 1.10
4122 reflections
188 parameters
H-atom parameters constrained
Δρ_max_ = 0.44 e Å^−3^
Δρ_min_ = −0.46 e Å^−3^

### 

Data collection: *CrystalClear* (Rigaku, 2005 ▶); cell refinement: *CrystalClear*; data reduction: *CrystalClear*; program(s) used to solve structure: *SHELXS97* (Sheldrick, 2008 ▶); program(s) used to refine structure: *SHELXL97* (Sheldrick, 2008 ▶); molecular graphics: *DIAMOND* (Brandenburg & Putz, 2005 ▶); software used to prepare material for publication: *SHELXL97*.

## Supplementary Material

Crystal structure: contains datablock(s) I, global. DOI: 10.1107/S1600536812021666/kp2409sup1.cif

Structure factors: contains datablock(s) I. DOI: 10.1107/S1600536812021666/kp2409Isup2.hkl

Additional supplementary materials:  crystallographic information; 3D view; checkCIF report

## Figures and Tables

**Table 1:** Selected bond lengths (Å)

Cl3—Sn1	2.4205 (11)
Cl4—Sn1	2.4076 (7)
Cl5—Sn1	2.4356 (7)

**Table 2:** Hydrogen-bond geometry (Å, °)

*D*—H⋯*A*	*D*—H	H⋯*A*	*D*⋯*A*	*D*—H⋯*A*
N1—H1*A*⋯Cl5^i^	0.89	2.64	3.522 (2)	172
N1—H1*A*⋯Cl3^i^	0.89	2.98	3.424 (2)	112
N1—H1*B*⋯Cl6^ii^	0.89	2.25	3.123 (3)	165
N1—H1*C*⋯Cl6^iii^	0.89	2.26	3.120 (3)	162
N2—H2*A*⋯Cl3^iv^	0.89	2.75	3.455 (2)	137
N2—H2*A*⋯Cl4^v^	0.89	2.79	3.567 (2)	147
N2—H2*A*⋯Cl4^iv^	0.89	2.92	3.344 (3)	111
N2—H2*B*⋯Cl6^vi^	0.89	2.20	3.085 (3)	175
N2—H2*C*⋯Cl5^vii^	0.89	2.61	3.424 (3)	153

Symmetry codes: (i) 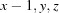; (ii) 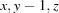; (iii) 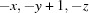; (iv) 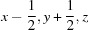; (v) 
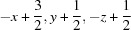
; (vi) 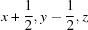; (vii) 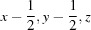.

**Fig. 1.**
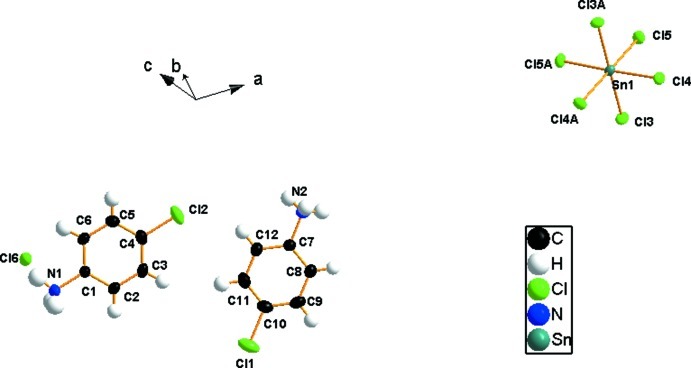
The molecular structure of the title compound with the atomic numbering scheme. Displacement ellipsoids are drawn at the 30% probability level.[The suffix A denotes the symmetry code: 2-x y 1/2 - z.]

**Fig. 2.**
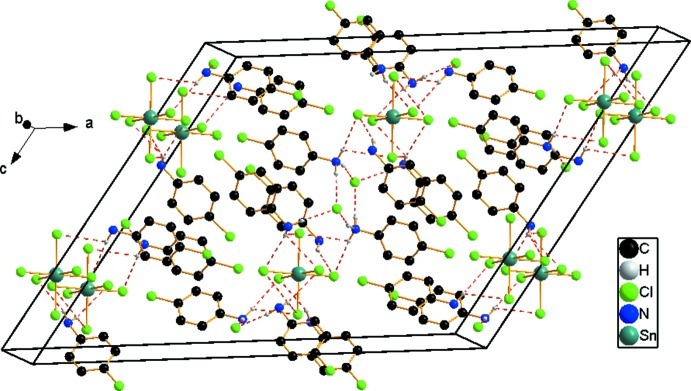
Crystal packing of the title compound. Dashed lines indicate hydrogen bonds.

## supplementary crystallographic information

### Comment

The study of ferroelectric materials has received much attention; some of them have predominantly dielectric–ferroelectric performance (Ye *et al.*, 2006; Fu *et al.*, 2007; Zhao *et al.* 2008; Zhang *et al.*, 2008; Ye *et al.*, 2009). As a part of our work to obtain potential ferroelectric phase-transition material, we report herein on the crystal structure of title compound. Unluckily, the title compound has no dielectric anomalies in the temperature range 93–453 K, suggesting that it might be only a paraelectric.

The asymmetric unit of the title compound is shown in Fig. 1 and Sn-Cl bonds are listed in Table 1. The crystal packing (Fig. 2) is stabilised by intermolecular N—H···Cl hydrogen bonds between the [C_6_H_7_ClN]^+^cations and SnCl_6_^2–^anions and Cl^-^ anions (Table 2).

### Experimental

For the preparation of the title compound, the water solution of the hydrochloric acid (10 mmol) was added to the ethanol solution of the 4-chlorobenzenamine(10 mmol), then the water solution of the SnCl_4_(5 mmol) was added into a reaction mixture. The resulting precipitate was filtered. Colourless crystals suitable for X-ray analysis were formed after several weeks by slow evaporation of the solvent at room temperature.

### Refinement

Positional parameters of all the H atoms bonded to C atoms were calculated geometrically and were allowed to ride on the C atoms to which they are bonded, with *U*_iso_(H) = 1.2Ueq(C) and *U*_iso_(H) = 1.5Ueq(C) for the methyl group. The other H bonded to N atoms were calculated geometrically and were allowed to ride on the N atoms with *U*_iso_(H) = 1.5Ueq(O).

### Crystal data

**Table:**

(C_6_H_7_ClN)_4_[SnCl_6_]Cl_2_	*F*(000) = 1816
*M**_r_* = 916.59	*D*_x_ = 1.692 Mg m^−^^3^
Monoclinic, *C*2/*c*	Mo *K*α radiation, λ = 0.71073 Å
Hall symbol: -C 2yc	Cell parameters from 4122 reflections
*a* = 27.855 (6) Å	θ = 3.4–27.5°
*b* = 7.2061 (14) Å	µ = 1.63 mm^−^^1^
*c* = 21.895 (4) Å	*T* = 293 K
β = 125.03 (3)°	Prism, colourless
*V* = 3598.8 (18) Å^3^	0.20 × 0.20 × 0.20 mm
*Z* = 4	

### Data collection

**Table:**

Rigaku SCXmini diffractometer	4122 independent reflections
Radiation source: fine-focus sealed tube	3581 reflections with *I* > 2σ(*I*)
Graphite monochromator	*R*_int_ = 0.031
Detector resolution: 13.6612 pixels mm^-1^	θ_max_ = 27.5°, θ_min_ = 3.4°
CCD_Profile_fitting scans	*h* = −36→35
Absorption correction: multi-scan (*CrystalClear*; Rigaku, 2005)	*k* = −9→9
*T*_min_ = 0.715, *T*_max_ = 0.730	*l* = −28→28
17870 measured reflections	

### Refinement

**Table:**

Refinement on *F*^2^	Primary atom site location: structure-invariant direct methods
Least-squares matrix: full	Secondary atom site location: difference Fourier map
*R*[*F*^2^ > 2σ(*F*^2^)] = 0.030	Hydrogen site location: inferred from neighbouring sites
*wR*(*F*^2^) = 0.067	H-atom parameters constrained
*S* = 1.10	*w* = 1/[σ^2^(*F*_o_^2^) + (0.0236*P*)^2^ + 4.0429*P*] where *P* = (*F*_o_^2^ + 2*F*_c_^2^)/3
4122 reflections	(Δ/σ)_max_ = 0.002
188 parameters	Δρ_max_ = 0.44 e Å^−^^3^
0 restraints	Δρ_min_ = −0.46 e Å^−^^3^

### Special details

**Table:**

Geometry. All e.s.d.'s (except the e.s.d. in the dihedral angle between two l.s. planes) are estimated using the full covariance matrix. The cell e.s.d.'s are taken into account individually in the estimation of e.s.d.'s in distances, angles and torsion angles; correlations between e.s.d.'s in cell parameters are only used when they are defined by crystal symmetry. An approximate (isotropic) treatment of cell e.s.d.'s is used for estimating e.s.d.'s involving l.s. planes.
Refinement. Refinement of *F*^2^ against ALL reflections. The weighted *R*-factor *wR* and goodness of fit *S* are based on *F*^2^, conventional *R*-factors *R* are based on *F*, with *F* set to zero for negative *F*^2^. The threshold expression of *F*^2^ > σ(*F*^2^) is used only for calculating *R*-factors(gt) *etc*. and is not relevant to the choice of reflections for refinement. *R*-factors based on *F*^2^ are statistically about twice as large as those based on *F*, and *R*- factors based on ALL data will be even larger.

### Fractional atomic coordinates and isotropic or equivalent isotropic displacement parameters (Å^2^)

**Table:**

	*x*	*y*	*z*	*U*_iso_*/*U*_eq_	
C1	0.12061 (12)	0.1500 (4)	0.12876 (15)	0.0503 (6)	
C2	0.13487 (13)	0.0639 (4)	0.08532 (16)	0.0540 (7)	
H2	0.1066	−0.0001	0.0425	0.065*	
C3	0.19188 (13)	0.0738 (4)	0.10633 (16)	0.0535 (7)	
H3	0.2025	0.0155	0.0779	0.064*	
C4	0.23257 (12)	0.1696 (4)	0.16906 (17)	0.0507 (7)	
C5	0.21803 (13)	0.2551 (5)	0.21162 (17)	0.0632 (8)	
H5	0.2463	0.3194	0.2544	0.076*	
C6	0.16093 (14)	0.2459 (5)	0.19102 (17)	0.0661 (9)	
H6	0.1503	0.3047	0.2195	0.079*	
C7	0.39010 (11)	0.0792 (3)	0.05568 (13)	0.0401 (5)	
C8	0.38139 (12)	−0.0100 (3)	−0.00526 (14)	0.0470 (6)	
H8	0.4121	−0.0253	−0.0103	0.056*	
C9	0.32612 (14)	−0.0767 (4)	−0.05904 (15)	0.0556 (7)	
H9	0.3192	−0.1366	−0.1010	0.067*	
C10	0.28179 (12)	−0.0544 (4)	−0.05054 (16)	0.0554 (7)	
C11	0.29063 (13)	0.0384 (5)	0.00988 (18)	0.0634 (8)	
H11	0.2597	0.0554	0.0144	0.076*	
C12	0.34538 (13)	0.1062 (4)	0.06368 (16)	0.0559 (7)	
H12	0.3519	0.1694	0.1049	0.067*	
Cl1	0.21316 (4)	−0.14614 (15)	−0.11590 (6)	0.0983 (3)	
Cl2	0.30443 (4)	0.17986 (14)	0.19641 (6)	0.0843 (3)	
Cl3	0.94108 (3)	0.11974 (9)	0.11503 (3)	0.04947 (16)	
Cl4	1.06231 (3)	−0.11341 (9)	0.25190 (4)	0.04712 (15)	
Cl5	1.06027 (3)	0.36499 (8)	0.24919 (4)	0.04846 (15)	
Cl6	0.02508 (4)	0.72478 (14)	0.05762 (5)	0.0808 (3)	
N1	0.05994 (11)	0.1377 (4)	0.10653 (15)	0.0707 (8)	
H1A	0.0577	0.1853	0.1423	0.106*	
H1B	0.0488	0.0193	0.0993	0.106*	
H1C	0.0365	0.2010	0.0645	0.106*	
N2	0.44908 (10)	0.1413 (3)	0.11539 (12)	0.0525 (5)	
H2A	0.4466	0.2420	0.1370	0.079*	
H2B	0.4692	0.1682	0.0964	0.079*	
H2C	0.4674	0.0515	0.1492	0.079*	
Sn1	1.0000	0.12149 (3)	0.2500	0.03220 (7)	

### Atomic displacement parameters (Å^2^)

**Table:**

	*U*^11^	*U*^22^	*U*^33^	*U*^12^	*U*^13^	*U*^23^
C1	0.0478 (15)	0.0652 (17)	0.0503 (15)	−0.0127 (13)	0.0353 (13)	−0.0134 (13)
C2	0.0628 (18)	0.0596 (17)	0.0540 (16)	−0.0140 (14)	0.0419 (15)	−0.0158 (13)
C3	0.0664 (18)	0.0497 (15)	0.0680 (18)	−0.0019 (13)	0.0524 (17)	−0.0038 (13)
C4	0.0492 (15)	0.0470 (15)	0.0678 (18)	0.0016 (12)	0.0405 (15)	0.0108 (13)
C5	0.0535 (17)	0.075 (2)	0.0566 (17)	−0.0164 (15)	0.0292 (15)	−0.0175 (15)
C6	0.0631 (19)	0.086 (2)	0.0632 (19)	−0.0188 (17)	0.0446 (17)	−0.0322 (17)
C7	0.0448 (13)	0.0332 (12)	0.0393 (12)	0.0021 (10)	0.0223 (11)	0.0041 (9)
C8	0.0551 (16)	0.0395 (13)	0.0508 (15)	0.0061 (11)	0.0329 (13)	0.0027 (11)
C9	0.072 (2)	0.0416 (14)	0.0429 (14)	−0.0030 (13)	0.0270 (15)	−0.0031 (11)
C10	0.0507 (16)	0.0471 (15)	0.0483 (15)	−0.0062 (12)	0.0167 (13)	0.0083 (12)
C11	0.0526 (17)	0.074 (2)	0.071 (2)	0.0020 (15)	0.0404 (17)	0.0082 (17)
C12	0.0602 (18)	0.0636 (18)	0.0546 (16)	0.0014 (14)	0.0392 (15)	−0.0034 (14)
Cl1	0.0635 (5)	0.0962 (7)	0.0859 (7)	−0.0256 (5)	0.0140 (5)	0.0026 (5)
Cl2	0.0541 (5)	0.0922 (6)	0.1164 (8)	0.0013 (4)	0.0548 (5)	0.0153 (6)
Cl3	0.0565 (4)	0.0537 (4)	0.0338 (3)	0.0105 (3)	0.0233 (3)	0.0055 (3)
Cl4	0.0442 (3)	0.0433 (3)	0.0505 (3)	0.0109 (3)	0.0252 (3)	−0.0019 (3)
Cl5	0.0575 (4)	0.0401 (3)	0.0623 (4)	−0.0089 (3)	0.0428 (3)	−0.0001 (3)
Cl6	0.0672 (5)	0.1076 (7)	0.0878 (6)	−0.0289 (5)	0.0563 (5)	−0.0438 (5)
N1	0.0547 (15)	0.110 (2)	0.0647 (16)	−0.0254 (14)	0.0443 (14)	−0.0329 (15)
N2	0.0499 (13)	0.0521 (13)	0.0477 (12)	0.0004 (10)	0.0234 (11)	−0.0010 (10)
Sn1	0.04091 (13)	0.02673 (11)	0.03437 (12)	0.000	0.02476 (10)	0.000

### Geometric parameters (Å, º)

**Table:**

C1—C6	1.356 (4)	C9—H9	0.9300
C1—C2	1.372 (4)	C10—C11	1.373 (4)
C1—N1	1.468 (3)	C10—Cl1	1.732 (3)
C2—C3	1.378 (4)	C11—C12	1.374 (4)
C2—H2	0.9300	C11—H11	0.9300
C3—C4	1.364 (4)	C12—H12	0.9300
C3—H3	0.9300	Cl3—Sn1	2.4205 (11)
C4—C5	1.359 (4)	Cl4—Sn1	2.4076 (7)
C4—Cl2	1.731 (3)	Cl5—Sn1	2.4356 (7)
C5—C6	1.383 (4)	N1—H1A	0.8900
C5—H5	0.9300	N1—H1B	0.8900
C6—H6	0.9300	N1—H1C	0.8900
C7—C12	1.368 (4)	N2—H2A	0.8900
C7—C8	1.371 (3)	N2—H2B	0.8900
C7—N2	1.464 (3)	N2—H2C	0.8900
C8—C9	1.381 (4)	Sn1—Cl4^i^	2.4076 (7)
C8—H8	0.9300	Sn1—Cl3^i^	2.4205 (11)
C9—C10	1.360 (4)	Sn1—Cl5^i^	2.4356 (7)
			
C6—C1—C2	121.7 (3)	C12—C11—H11	120.1
C6—C1—N1	119.7 (2)	C7—C12—C11	118.9 (3)
C2—C1—N1	118.6 (2)	C7—C12—H12	120.5
C1—C2—C3	118.8 (3)	C11—C12—H12	120.5
C1—C2—H2	120.6	C1—N1—H1A	109.5
C3—C2—H2	120.6	C1—N1—H1B	109.5
C4—C3—C2	119.6 (3)	H1A—N1—H1B	109.5
C4—C3—H3	120.2	C1—N1—H1C	109.5
C2—C3—H3	120.2	H1A—N1—H1C	109.5
C5—C4—C3	121.2 (3)	H1B—N1—H1C	109.5
C5—C4—Cl2	119.0 (2)	C7—N2—H2A	109.5
C3—C4—Cl2	119.9 (2)	C7—N2—H2B	109.5
C4—C5—C6	119.6 (3)	H2A—N2—H2B	109.5
C4—C5—H5	120.2	C7—N2—H2C	109.5
C6—C5—H5	120.2	H2A—N2—H2C	109.5
C1—C6—C5	119.1 (3)	H2B—N2—H2C	109.5
C1—C6—H6	120.5	Cl4^i^—Sn1—Cl4	90.65 (4)
C5—C6—H6	120.5	Cl4^i^—Sn1—Cl3	89.91 (3)
C12—C7—C8	121.7 (2)	Cl4—Sn1—Cl3	89.67 (3)
C12—C7—N2	118.7 (2)	Cl4^i^—Sn1—Cl3^i^	89.67 (3)
C8—C7—N2	119.6 (2)	Cl4—Sn1—Cl3^i^	89.91 (3)
C7—C8—C9	118.7 (3)	Cl3—Sn1—Cl3^i^	179.40 (3)
C7—C8—H8	120.6	Cl4^i^—Sn1—Cl5	178.18 (2)
C9—C8—H8	120.6	Cl4—Sn1—Cl5	90.77 (3)
C10—C9—C8	119.9 (3)	Cl3—Sn1—Cl5	88.97 (3)
C10—C9—H9	120.1	Cl3^i^—Sn1—Cl5	91.46 (3)
C8—C9—H9	120.1	Cl4^i^—Sn1—Cl5^i^	90.77 (3)
C9—C10—C11	121.0 (3)	Cl4—Sn1—Cl5^i^	178.18 (2)
C9—C10—Cl1	120.0 (2)	Cl3—Sn1—Cl5^i^	91.46 (3)
C11—C10—Cl1	119.0 (3)	Cl3^i^—Sn1—Cl5^i^	88.97 (3)
C10—C11—C12	119.7 (3)	Cl5—Sn1—Cl5^i^	87.82 (4)
C10—C11—H11	120.1		
			
C6—C1—C2—C3	0.8 (5)	C12—C7—C8—C9	−1.1 (4)
N1—C1—C2—C3	−179.5 (3)	N2—C7—C8—C9	176.5 (2)
C1—C2—C3—C4	−0.5 (4)	C7—C8—C9—C10	−0.5 (4)
C2—C3—C4—C5	0.3 (5)	C8—C9—C10—C11	1.9 (4)
C2—C3—C4—Cl2	179.1 (2)	C8—C9—C10—Cl1	−177.3 (2)
C3—C4—C5—C6	−0.3 (5)	C9—C10—C11—C12	−1.6 (5)
Cl2—C4—C5—C6	−179.1 (3)	Cl1—C10—C11—C12	177.6 (2)
C2—C1—C6—C5	−0.8 (5)	C8—C7—C12—C11	1.4 (4)
N1—C1—C6—C5	179.5 (3)	N2—C7—C12—C11	−176.2 (3)
C4—C5—C6—C1	0.5 (5)	C10—C11—C12—C7	0.0 (5)

Symmetry code: (i) −*x*+2, *y*, −*z*+1/2.

### Hydrogen-bond geometry (Å, º)

**Table:**

*D*—H···*A*	*D*—H	H···*A*	*D*···*A*	*D*—H···*A*
N1—H1*A*···Cl5^ii^	0.89	2.64	3.522 (2)	172
N1—H1*A*···Cl3^ii^	0.89	2.98	3.424 (2)	112
N1—H1*B*···Cl6^iii^	0.89	2.25	3.123 (3)	165
N1—H1*C*···Cl6^iv^	0.89	2.26	3.120 (3)	162
N2—H2*A*···Cl3^v^	0.89	2.75	3.455 (2)	137
N2—H2*A*···Cl4^vi^	0.89	2.79	3.567 (2)	147
N2—H2*A*···Cl4^v^	0.89	2.92	3.344 (3)	111
N2—H2*B*···Cl6^vii^	0.89	2.20	3.085 (3)	175
N2—H2*C*···Cl5^viii^	0.89	2.61	3.424 (3)	153

Symmetry codes: (ii) *x*−1, *y*, *z*; (iii) *x*, *y*−1, *z*; (iv) −*x*, −*y*+1, −*z*; (v) *x*−1/2, *y*+1/2, *z*; (vi) −*x*+3/2, *y*+1/2, −*z*+1/2; (vii) *x*+1/2, *y*−1/2, *z*; (viii) *x*−1/2, *y*−1/2, *z*.
